# The influence of different THA surgical approaches on Patient’s early postoperative anxiety and depression

**DOI:** 10.1186/s12891-021-04746-z

**Published:** 2021-10-08

**Authors:** Tianshu Shi, Qianjin Wang, Siyu Shen, Yong Shi, Jian Huang, Ke Lu, Qing Jiang

**Affiliations:** 1 State Key Laboratory of Pharmaceutical Biotechnology, Department of Sports Medicine and Adult Reconstructive Surgery, Nanjing Drum Tower Hospital, The Affiliated Hospital of Nanjing University Medical School, 321 Zhongshan Road, Nanjing, 210008 Jiangsu PR China; 2 Laboratory for Bone and Joint Disease, Model Animal Research Center (MARC), Nanjing University, Nanjing, 210093 Jiangsu PR China; 3 Department of Orthopedic Surgery, Rush University Medical Center, Chicago, IL 60612 USA

**Keywords:** OCM, DAA, Depression, Anxiety, Psychological disease

## Abstract

**Introduction:**

Total hip arthroplasty (THA) is generally considered to be one of the most successful orthopedic surgical procedures. However, no research has been conducted on the postoperative mental health of patients who underwent different approaches of THA. This paper seeks to compare the differences among three THA approaches: the normal lateral approach (NLA), the direct anterior approach (DAA) and the orthopädische chirurgie münchen (OCM) regarding their influence on patients’ postoperative anxiety and depression.

**Method:**

A total of 95 THA patients were recruited for this study. All patients’ preoperative information including results of Harris, SF-36 and Visual Analogue Scale (VAS) was carefully evaluated. Surgery-related data as well as five-day postoperative data were also collected. Three months after the surgery, a telephone follow-up was conducted to further evaluate patients’ HADS and SF-36 results.

**Result:**

In the three-month postoperative evaluation of anxiety and depression, the NLA group scored significantly higher than both the DAA group and the OCM group, which was found relevant to the patient’s incision length and five-day postoperative VAS results. A correlation between anxiety scores and the days of postoperative hospitalization was also noticed. Further analysis of patients’ psychological state based on the SF-36 results revealed considerable differences in viability (VT) and social function (SF) between the NLA group and the OCM group. Other surgery-related data and postoperative data all demonstrated better results of the DAA group and the OCM group compared to the NLA group.

**Conclusion:**

Among the three different surgical approaches of THA, DAA and OCM compared with NLA are found to ease patients’ postoperative anxiety and depression.

**Level of evidence:**

**III**

## Introduction

The hip is the largest weight-bearing joint and also one of the most vulnerable to injury. Various diseases, including hip osteoarthritis, osteonecrosis of femoral head and developmental dysplasia of the hip (DDH), severely affect the hip’s range of motion [1, 2]. Total hip arthroplasty (THA), intended to alleviate pain and restore physiological mobility, has been one of the most revolutionary surgical treatments since the late 20th and early 21st centuries [3].

The normal lateral approach (NLA) is the most commonly used surgical approach in THA surgeries globally. However, a small number of patients continue to report symptoms, usually pain, even after the NLA procedure. Possible explanations include damages to muscles, tendons and their innervations associated with traumatic reactions to the surgical procedure [1]. Over the past decades, the technique of “minimally invasive surgery (MIS)”, which focuses on decreasing surgical incision length and minimizing muscular trauma, has been developed greatly [2]. As a result, the direct anterior approach (DAA) and the orthopädische chirurgie münchen (OCM) [4, 5], two MIS procedures of the THA surgeries, have been steadily gaining popularity given their low risk of dislocation, less muscular damage, potential for earlier function recovery as well as improvement of patients’ satisfaction [6–8]. Both DAA and OCM, together with NLA, were included in this study.

Aside from patients’ physical conditions, it is also necessary to include mental health when considering patients’ overall well-being. Several factors, such as the length of incision, duration of rehabilitation, and pain management, have been shown to have great influence on patients’ postoperative psychological status, including possible occurrence of anxiety and depression [9]. Moreover, previous researches showed the pain controlling used the radiation therapy or drugs could ensuring of local disease during bone metastasis surgery, which may contribute to alleviate the anxiety and depression during bone metastasis [10–12]. Generally, anxiety, depression and other related psychological symptoms exert negative effects on patients’ motivation, spirit, and ability to cope with illness and overall adherence [13]. Therefore, psychological symptoms are highly worth paying attention to when assessing treatment results and rehabilitation following the THA surgery.

Based on the above information, the purpose of this study was to compare different psychological influences of THA surgery performed through OCM, DAA or NLA, and to determine possible factors associated with postoperative anxiety and depression.

## Method

### Participants

**Fig. 1:**
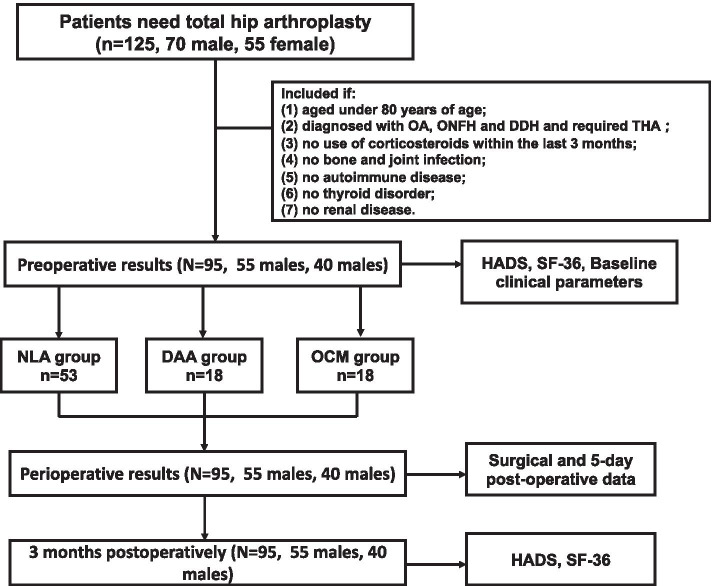
Flow chart of patient allocation and participation. OCM: the orthopädische chirurgie münchen; NLP: The normal lateral approach; DAA: the direct anterior approach

In this study, all participants were enrolled from patients hospitalized from November 2015 to May 2016 in Department of Sports Medicine & Adult Reconstructive Surgery at Nanjing Drum Tower Hospital (the affiliated hospital of Nanjing University Medical School). We included patients aged under 80 years and diagnosed with OA, ONFH or DDH treated with THA in this study. The exclusion criteria were as follows:(1) use of corticosteroids within the last 3 months; (2) bone and jiont infections; (3) autoimmune disease; (4) thyroid disorder; (5) renal disease. In the end, a total of 95 participants were enrolled in this study. The study was approved by the ethics committee of Drum Tower Hospital (No.: 2020–350), and all patients provided written informed consent (Fig. 1).

### Surgical approach

All participants enrolled in this study underwent THA surgery through NLA, DAA or OCM randomly. There was no specific indication for the approach for THAs. All surgeries were randomly performed by three fully qualified and experienced orthopaedic surgeons. Brief introductions to the three surgery approaches are as follows:

The NLA: The patient was in the lateral decubitus position. The 10–15 cm-long incision was started near the tip of the trochanter and extended in line with the femur, forming a curved incision from the tip to the mid-trochanter line. Then the skin and subcutaneous fat were incised down to the fascia, and the fascia was incised accordingly. After carefully identifying the fiber orientation of the gluteus medius muscle and the lateral femoral muscle, incision was started from the anterior third of the gluteus medius muscle and extended to the lateral femoral muscle. Next the joint capsule was cut open and the hip joint was dislocated by external rotation. After finishing replacement of all components, the wound was closed.

The DAA: The patient was in the supine position. The 10 cm-long incision was started 3 cm lateral and distal to the anterior superior iliac spine and then continued along the muscles to the tensor fasciae latae. A longitudinal incision was then made along the interval between the tensor fasciae latae and the sartorius muscles to create an inter-muscular portal and then blunt dissection medial to the tensor fasciae latae was performed. After deep dissection to expose the anterior capsule, femoral neck cut was made and the acetabulum was then exposed, followed by acetabular preparation and acetabular component implantation. Then femoral exposure was performed, followed by femoral preparation, trailing, and component implantation. When replacement was finished, the wound was closed.

The OCM: The patient was in the lateral decubitus position. Skin incision was made over the anterior portion of the greater trochanter, which was slightly curved and then continued over the muscular interval between gluteus medius and tensor fasciae latae. Fascia incision was performed, followed by exposure and incision of capsule. Then acetabular and femoral components were implanted accordingly. After replacing all the components, the wound was closed.

### Data collection

Before surgery, all recruited participants were collected the basic information, including the gender, age, weight, height, body mass index (BMI), the reasons for total hip arthroplasty (THA). Moreover, the results of MOS 36-item short-form health survey (SF-36) were analyzed, which could assess the healthy statue of patients, including physical functioning (PF), role-physical (RP), bodily pain (BP), general health (GH), vitality (VT), social function (SF), role-emotion (RE) and mental health (MH). Harris score (Harris hip score) and Visual Analogue Scale (VAS) results were also collected to evaluate the patient’s pre-operative hip functions as well as the scale of pain. Routine tests of biochemical levels including ESR, CRP and D-Dimer were conducted both before and after the surgery. Information related to the surgical process and post-operative conditions was also recorded, including time of operation, amount of bleeding, incision length and time of admission and discharge. A telephone follow-up without knowing the operation details before was conducted three months after the operation to further evaluate patients’ HADS and SF-36 results.

### Statistical analysis

All the statistical analysis was performed using SPSS v22.0 (SPSS Inc., Chicago, IL, USA) and graphs were done with GraphPad Prism v7.0 (GraphPad Software Inc., La Jolla, CA, USA). Results were expressed as means ± SD. Data were compared across different approaches with a one-way ANOVA test, where indicated Pearson correlation was used to evaluate the relationship between different variables. A *p* value smaller than 0.05 was considered statistically significant.

## Result

### Baseline clinical parameters

A total of 95 patients treated with THA (45 males, 50 female) were involved in this study. Mean age ± SD for the NLA, DAA and OCM groups was 63.6 ± 8.93, 58.9 ± 7.21 and 56.8 ± 8.17 respectively. Also, the BMI for all three groups was 25.8 ± 2.49, 23.95 ± 2.45 and 23.04 ± 3.06 accordingly. Differences regarding these two variables were noticed between the NLA and the OCM groups, aside from which no further differences were found significant. (Table 1). Also, based on the tests conducted when admitting the recruited patients, all three groups showed no significant differences regarding serology, Harris results and all other parameters (Table 2).

**Table 1:** Sample Variables and Group Distribution

	NLA	DAA	OCM	*P*
Sex (male/female)(F%)	24/29 (54.7%)	10/8 (44.4%)	11/13 (54.1%)	
Age (years)	63.6 ± 8.93	58.9 ± 7.21	56.8 ± 8.17	N vs O *
Weight (kg)	64.5 ± 4.6	61.4 ± 10.19	61.2 ± 8.21	–
Height (cm)	161.0 ± 5.41	162.8 ± 4.93	164.0 ± 7.39	–
BMI (kg/m2)	25.8 ± 2.49	23.95 ± 2.45	23.04 ± 3.06	N vs O *
Hip OA	4	3	5	
Femoral head necrosis	34	11	13	
DDH	15	4	6	

Values are expressed as mean ± standard deviation (range) *, *p* < 0.05; **, *p* < 0.01; ***, *p* < 0.0001

*OCM* The orthopädische chirurgie münchen; *NLP* The normal lateral approach; *DAA* The direct anterior approach; *DDH* The developmental dislocation of the hip

**Table 2:** Preoperative Scores and Serum index

	NLA	DAA	OCM	*P*
Harris score	46.85 ± 11.28	45.17 ± 6.42	44.47 ± 7.05	–
VAS score (mm)	56.73 ± 14.62	56.95 ± 13.42	57.92 ± 13.13	–
PCS-SF-36				
PF	23.21 ± 8.22	23.75 ± 5.82	24.06 ± 8.2	–
RP	47.67 ± 30.17	42.5 ± 16.48	50 ± 23.13	–
BP	35.65 ± 23.33	37.5 ± 29.83	40.82 ± 30.87	–
GH	41.76 ± 16.48	46.18 ± 16.25	46.76 ± 11.17	–
MCS-SF-36				
VT	52.5 ± 32.45	44.26 ± 33.57	48.82 ± 34.59	–
SF	58 ± 30.75	52.81 ± 26.58	61.18 ± 32.76	–
RE	67.94 ± 24.01	69.82 ± 26.43	72.5 ± 25.11	–
MH	57.2 ± 22.98	61 ± 22.43	60.5 ± 23.14	–
ESR (mm/h)	14.66 ± 10.21	18 ± 13.6	16.22 ± 9.57	–
CRP (mg/L)	5.12 ± 2.59	7.25 ± 3.41	5.24 ± 2.64	–
D-Dimer (mg/L)	0.4 ± 0.22	0.46 ± 0.23	0.34 ± 0.17	–
HGB(g/L)	136.2 ± 13.86	140.3 ± 10.36	134.3 ± 11.99	–
HCT(%)	40.79 ± 3.9	41.61 ± 2.8	40.32 ± 3.12	–

Values are expressed as mean ± standard deviation (range)*, *p* < 0.05; **, *p* < 0.01; ***, *p* < 0.0001

*VAS* Visual analogue scale, *PF* Physical Functioning, *RP* Role-Physical, *BP* Bodily Pain, *GH* General Health, *VT* Vitality, *SF* Social Functioning, *RE* Role-Emotional, *MH* Mental Health, *ESR* Erythrocyte Sedimentation Rate, *CRP* C-Reactive Protein, *HGB* Hemoglobin, *HCT* Hematocrit

### Three-month postoperative score changes

In order to look into the psychological impact of different THA approaches, a telephone follow-up was conducted three months after the operation. The anxiety score of the HADS was significantly higher in the NLA group than the DAA and OCM groups by 62.9 and 77.2% respectively. As for the depression part of HADS, the NLA group scored higher than the DAA and OCM groups by 55.3 and 78.4% respectively. In the comparison of pre-operative and post-operative SF-36 results, the post-operative results were higher in all aspects covered. Nevertheless, differences between the three groups still existed. When it came to the physical function (PF) scores of the physical component summary scale (PCS), the OCM groups scored higher than the NLA group by 22.5%. A similar trend was noticed in the score of general health (GH), where the results of the OCM group were 29.5% higher than those of the NLA group. Furthermore, in the mental component summary scale, the OCM groups again scored 21.6% higher in vitality (VT) and 32% higher in social function (SF) compared to the NLA group. No significant differences regrading other aspects were noticed among the three groups. (Table 3).

**Table 3:** 3 months Postoperative Scores

	NLA	DAA	OCM	*P*
HADS-A	6.23 ± 4.69	2.28 ± 1.7	1.42 ± 1.36	N vs D* N vs O****
HADS-D	5.37 ± 3.07	2.4 ± 1.57	1.16 ± 1.04	N vs D** N vs O****
PCS-SF-36				
PF	51.93 ± 15.89	54.3 ± 9.03	65.2 ± 12.06	N vs O *
RP	83.13 ± 16.72	75 ± 40.09	86.9 ± 28.3	–
BP	76.6 ± 8.9	84.18 ± 16.18	86.44 ± 15.62	–
GH	55.29 ± 16.25	64.38 ± 20.35	71.67 ± 15.62	N vs O*
MCS-SF-36				
VT	62.09 ± 21.87	65.6 ± 25.8	76.5 ± 21	N vs O *
SF	61.43 ± 28.78	73.7 ± 22	81.1 ± 18.1	N vs O *
RE	81.36 ± 17.85	87.3 ± 24.9	88.8 ± 25.9	–
MH	67.73 ± 19.4	81.71 ± 13.73	76.71 ± 17.59	–

Values are expressed as mean ± standard deviation (range) *, *p* < 0.05; **, *p* < 0.01; ***, *p* < 0.0001

*HADS* Hospital Anxiety and Depression Scale

### Surgical and 5-day post-operative data changes

The different influences of all three approaches on patients’ post-operative anxiety and depression led to further research into the possible causes. The physiological and biochemical indicators during and after the surgery were carefully evaluated, revealing that the surgery time of the OCM group was 20.7% shorter than that of the NLA group. The surgery time of DAA group was slightly longer than the NLA group by 5.4%. Differences of total hospitalization time and post-operative hospitalization time were also noticed. Patients of the OCM group overall spent relatively shorter time in hospital than those of the NLA group (25.13%) and the DAA group (32.9%). The total hospitalization time spent by the DAA group was slightly longer than the NLA group (11.5%). As for post-operative hospitalization time, the OCM group also showed a 23.9% decrease compared to the NLA group, but no significant difference was found compared to the DAA group. Of all aspects, the most noticeable difference occurred in the incision length. The incision length of the OCM group was 33.6% shorter than that of the NLA group and 12.4% shorter compared to the DAA group. Also, a 24.2% increase of incision length was noticed in the NLA group when compared to the DAA group. Other differences include 5-day post-operative blood CRP levels and D-dimer levels, where the results of the OCM groups were 43.11 and 41.23% lower than those of the NLA and DAA group respectively. On top of all the above aspects, statistics related to the nursing of patients on the 5th post-operative day showed that the OCM group did much better than the NLA group. The NLA group scored 22.5% lower than the OCM group in ADL, and the Barthel score of the OCM group was 6.5% higher than that of the NLA group. As for the VAS evaluation, it was also discovered that the OCM group scored 43.8% lower than the NLA group. No significant differences regrading other post-operative aspects were noticed (Table 4).

**Table 4:** Surgical and 5-days Post-Operative Data

	NLA	DAA	OCM	*P*
Operative time (min)	105.9 ± 24.9	111.6 ± 16.93	83.93 ± 19.33	N vs O ** D vs O *
Blood loss (ml)	292.6 ± 164	211.8 ± 139.8	287.1 ± 138.2	–
Hospital stay (Days)	11.46 ± 3.85	12.78 ± 2.27	8.58 ± 2.67	N vs O * D vs O *
Post-operative time (Days)	6.88 ± 2.16	6.89 ± 1.05	5.23 ± 1.39	N vs O **
Incision length (mm)	12.76 ± 1.087	9.67 ± 0.51	8.47 ± 0.56	N vs D**** N vs O**** *D* vs O *
ESR (mm/h)	51.97 ± 19.88	41.88 ± 18.52	43.5 ± 21.3	–
CRP (mg/L)	52.25 ± 32.57	39.17 ± 23.1	29.72 ± 18.67	N vs O *
D-Dimer (mg/L)	3.59 ± 2.1	3.75 ± 2.4	2.11 ± 1.19	N vs O *
HGB(g/L)	106.2 ± 13.47	113.1 ± 9.43	104 ± 14.1	–
HCT(%)	31.7 ± 3.82	33.3 ± 3.07	30.9 ± 4.03	–
Braden Score	15.35 ± 0.93	15.33 ± 0.7	15.47 ± 0.71	–
ADL score	25.51 ± 4.23	26.67 ± 3.53	31.25 ± 8.09	N vs O ***
Barthel score	85.53 ± 8.38	84.4 ± 6.82	91.1 ± 7.25	N vs O **
VAS	11.51 ± 6.62	12.2 ± 6.66	6.47 ± 4.9	N vs O *

Values are expressed as mean ± standard deviation (range) *, *p* < 0.05; **, *p* < 0.01; ***, *p* < 0.0001

*ADL* Activities of Daily Living

### Correlation between HADS scores and operative data

Based on the above results, we conducted an analysis of the correlation between HADS scores and both the operative and post-operative data. Strong correlations were found between the anxiety score of HADS and post-operative hospitalization time (*r* = 0.3994, *p* = 0.0158), incision length (*r* = 0.4333, *p* = 0.0105) and 5-day post-operative pain scale of VAS (*r* = 0.3498, *p* = 0.0338). Also it was noticed that the depression score correlated with only the incision length (*r* = 0.3467, *p* = 0.0446) and 5-day post-operative pain scale of VAS (*r* = 0.4122, *p* = 0.0082). Variations of other post-operative parameters demonstrated no strong influence on HADS scores (Table 5).

**Table 5:** Pearson and Spearman correlations

	HADS-A		HADS-D	
Operative time	*r* = 0.143	*–*	*r* = 0.0148	*–*
Blood loss	*r* = −0.1698	*–*	*r* = −0.0396	–
Hospital stay	*r* = 0.264	*–*	*r* = 0.2799	*–*
Post-oprative time	*r* = 0.3994	*p =* 0.0158*	*r* = 0.1979	–
Incision length	*r* = 0.4333	*p =* 0.0105*	*r* = 0.3467	*p =* 0.0446*
ESR (mm/h)	*r* = 0.103	*–*	*r* = 0.155	*–*
CRP (mg/L)	*r* = 0.3444	*–*	*r* = 0.3313	–
D-Dimer (mg/L)	*r* = −0.1542	*–*	*r* = 0.04986	*–*
Braden Score	*r* = −0.174	–	*r* = −0.04458	–
ADL score	*r* = 0.143	*–*	*r* = 0.04376	*–*
Barthel	*r* = −0.1516	–	*r* = −0.1739	–
VAS	*r* = 0.3498	*p =* 0.0338*	*r* = 0.4122	*p =* 0.0082**

*, *p* < 0.05; **, *p* < 0.01; ***, *p* < 0.0001

## Discussion

In our study, we discovered that three THA approaches exerted significantly different influences on patients’ 3-month post-operative psychological indexes. The DAA and OCM groups scored relatively lower than the NLA group in anxiety and depression scores of the HADS. Based on conclusions of the SF-36 results, possible psychological differences occurred in patients’ vitality (VT) and social function (SF), which was likely to correlate with shorter incision length and lower VAS pain scales of the DAA and OCM groups.

In fact, anxiety refers to individuals’ relatively stable perceptions in stressful situations and reflects individual differences in sensitivity to a negative or threatening stimulus [14]. And depression is generally considered to be a more serious mental state regarding the stressful situations. Many potential factors can contribute to the hospitalized patients’ anxiety and depression, including pain, pre-operative education level, length of lower limbs, levels of relief, etc. [15, 16]. Maaike M. Vissers et.al. discovered that certain psychological status like anxiety and depression dramatically affected operation results and satisfaction levels of total knee replacement patients, and that post-operative function recovery and pain control was highly correlated with depression [17]. Our study also revealed that great difference of patients’ 5-day post-operative pain levels existed between the NLA group and the OCM group (*p* < 0.05). Correlation analysis indicated that post-operative pain feeling was strongly correlated with post-operative anxiety and depression (p < 0.05 and *p* < 0.01, respectively). The result was consistent with Duivenvoorden et.al.’s report that when pain and disability decreases after THA or TKA, the prevalence of psychological symptoms also decreases [13]. However, our evaluation scales may not fully reflect the postoperative situation of patients [18]. Meccariello L et.al. used another clinical scale which concluded the psychological status of patient, which may could be used in our further studies [19].

Nowadays development of THA approach puts more emphasis on the minimal invasion of incision and the decreasing of muscle cut, taking advantage of the muscle and nerve interface to reduce separation of muscles and tendons [2]. The two minimally invasive approach used in this study were direct anterior approach (DAA) and the orthopädische chirurgie münchen (OCM). Both the approaches used blunt dissection of muscle intervals to separate and reveal the acetabulum and proved to be beneficial for early post-operative function recovery, pain relief and change of gait in patients. Early post-operative function recovery then contributed to the decreasing of patients’ anxiety and depression levels. In the post-operative SF-36 results collected in our study, PF scores varied significantly among three groups, which was possibly related to the patients’ post-operative psychological status. Meanwhile the analysis of operative data demonstrated that the OCM group showed better results than the NLA group regarding surgery time, post-operative hospitalization time and incision length. Further analysis then revealed correlation between incision length and post-operative anxiety as well as post-operative hospitalization time and post-operative anxiety (*p* = 0.0105 and *p* = 0.0108, respectively), on top of which correlation between incision length and depression was also determined (*p* = 0.0446). The results were consistent with the report of Ottokar Stundner et.al. Possible explanation includes that the area of tissue damage and short-term post-operative recovery level varied in different groups, which in turn affected the patients’ overall health conditions [15].

It was also noticed in our study that in NLA and OCM groups the 5-day post-operative CRP and D-dimer levels varied greatly, which was likely to be caused by the different effects of the approaches on patient’s bodies. In fact, the level of CRP and D-dimer were associated with the postoperative infection. However, according to our following observation, those patients collected in our studies have no severe complication [20, 21]. CRP was considered to correlate with patient’s post-operative local tissue inflammation, which indicates that OCM most mildly affects patients’ inflammation reaction [22, 23]. Although no correlation was determined between post-operative CRP levels and psychological indexes, the increase of inflammation was highly likely to aggravate patients’ anxiety and depression. D-dimer is a fibrin degradation product and can be used as a marker to reflect the degradation function of fibrin. D-dimer is associated with post-operative occurrence of thrombus [24]. In our study, the 5-day post-operative level of D-dimer in the OCM group was significantly lower than that of the NLA group, which could possibly be explained by different incision length and whether or not muscle cut was involved. Though the difference in D-dimer levels was not found relevant to patients’ psychological status, the increase of D-dimer indeed prolonged patients’ stay in the hospital and thus potentially laid more stress on patients, leading to possible increase of post-operative anxiety [25].

However, several limitations do exist in our study. Firstly, this study was based only at one center and the sample size was not big enough. Besides, the analysis in our study only relied on 3-month post-operative data, while data collected at a longer time after operation would be of more significance for further analysis of the three approaches’ psychological influences.

In conclusion, our study showed that compared to the normal lateral approach, minimally invasive surgery approaches (OCM, DAA) had less negative influence on patients’ post-operative anxiety and depression, which offers a new insight for clinical doctors when choosing the surgical approach for total hip arthroplasty.

## Data Availability

The datasets used and/or analysed during the current study are available from the corresponding author on reasonable request.

## Abbreviations

OCM: The orthopädische chirurgie münchen
NLP: The normal lateral approach
MIS: Minimally invasive surgery
DAA: The direct anterior approach
DDH: The developmental dislocation of the hip
CRP: C-reactive protein
VAS: Visual analogue scale
Harris: Harris hip score
PF: Physical Functioning
RP: Role-Physical
BP: Bodily Pain
GH: General Health
VT: Vitality
SF: Social Functioning
RE: Role-Emotional
MH: Mental Health;
ESR: Erythrocyte Sedimentation Rate
CRP: C-Reactive Protein
HGB: Hemoglobin
HCT: Hematocrit
HADS: Hospital Anxiety and Depression Scale
